# The “Great Debate” at Melanoma Bridge 2022, Naples, December 1st–3rd, 2022

**DOI:** 10.1186/s12967-023-04100-y

**Published:** 2023-04-18

**Authors:** Paolo A. Ascierto, Christian Blank, Alexander M. Eggermont, Claus Garbe, Jeffrey E. Gershenwald, Omid Hamid, Axel Hauschild, Jason J. Luke, Janice M. Mehnert, Jeffrey A. Sosman, Hussein A. Tawbi, Mario Mandalà, Alessandro Testori, Corrado Caracò, Iman Osman, Igor Puzanov

**Affiliations:** 1 Department of Melanoma, Cancer Immunotherapy and Innovative Therapy, Istituto Nazionale Tumori IRCCS “Fondazione G. Pascale”, Naples, Italy; 2 Netherlands Cancer Institute, Amsterdam, The Netherlands; 3 University Medical Center Utrecht & Princess Maxima Center, Utrecht, The Netherlands; 4 Comprehensive Cancer Center München, Technical University München & Ludwig Maximiliaan University, München, Germany; 5 Center for Dermatooncology, Department of Dermatology, Eberhard Karls University, Tuebingen, Germany; 6 Department of Surgical Oncology, The University of Texas MD Anderson Cancer Center, Houston, TX USA; 7 The Angeles Clinic and Research Institute, A Cedars-Sinai Affiliate, Los Angeles, CA USA; 8 Department of Dermatology, University of Kiel, Kiel, Germany; 9 University of Pittsburgh Medical Center (UPMC) Hillman Cancer Center, Pittsburgh, PA USA; 10 Perlmutter Cancer Center of NYU Langone/NYU Grossman School of Medicine, New York, NY USA; 11 Robert H Lurie Comprehensive Cancer Center, Northwestern University Medical Center, Chicago, IL USA; 12 MD Anderson Brain Metastasis Clinic UT, MD Anderson Cancer Center, Houston, TX USA; 13 University of Perugia, Perugia, Italy; 14 Image regenerative clinic Milan, Italy; EORTC Melanoma Group, Brussels, Belgium; 15 Division of Surgery of Melanoma and Skin Cancer, Istituto Nazionale Tumori “Fondazione Pascale” IRCCS, Naples, Italy; 16 Rudolf L. Baer, NYU Langone Medical Center, New York, NY USA; 17 Department of Medicine, Roswell Park Comprehensive Cancer Center, Buffalo, NY USA

**Keywords:** Melanoma, Immunotherapy, Anti-PD-1, Targeted therapy, Adjuvant, Neoadjuvant, Surgery

## Abstract

The Great Debate session at the 2022 Melanoma Bridge congress (December 1–3) featured counterpoint views from leading experts on five contemporary topics of debate in the management of melanoma. The debates considered the choice of anti-lymphocyte-activation gene (LAG)-3 therapy or ipilimumab in combination with anti-programmed death (PD)-1 therapy, whether anti-PD-1 monotherapy is still acceptable as a comparator arm in clinical trials, whether adjuvant treatment of melanoma is still a useful treatment option, the role of adjuvant therapy in stage II melanoma, what role surgery will continue to have in the treatment of melanoma. As is customary in the Melanoma Bridge Great Debates, the speakers are invited by the meeting Chairs to express one side of the assigned debate and the opinions given may not fully reflect personal views. Audiences voted in favour of either side of the argument both before and after each debate.

## Introduction

The Great Debate session at the 2022 Melanoma Bridge congress (December 2–3) featured counterpoint views from leading experts on five contemporary topics of debate in the management of melanoma. The debates considered the choice of anti-lymphocyte-activation gene (LAG)-3 therapy or ipilimumab in combination with anti-programmed death (PD)-1 therapy, whether anti-PD-1 monotherapy is still acceptable as a comparator arm in clinical trials, whether adjuvant treatment of melanoma is still a useful treatment option, the role of adjuvant therapy in stage II melanoma, what role surgery will continue to have in the treatment of melanoma. As is customary in the Melanoma Bridge Great Debates, the speakers are invited by the meeting Chairs to express one side of the assigned debate and the opinions given may not fully reflect personal views. Audiences voted in favour of either side of the argument both before and after each debate.

## LAG-3 versus ipilimumab sequencing

### Hussein A. Tawbi: in favour of LAG-3

The combination of nivolumab and ipilimumab was assessed in the phase III CheckMate 067 trial of 945 patients with previously untreated unresectable stage III/IV melanoma. After a follow-up 6.5 years, combination treatment with nivolumab 1 mg/kg plus ipilimumab 3 mg/kg or nivolumab alone was associated with significantly better overall response rate (ORR) and median overall survival (OS) than ipilimumab alone [1]. Improved survival was also seen with the combination compared with nivolumab alone, although this was just a descriptive analysis. Combination therapy was also associated with increased toxicity (59% of patients with a grade 3–4 adverse event versus 24% with nivolumab alone), the burden of which may be under-represented given that many patients experience more than one high-grade adverse event. However, anti-PD-1 therapy in combination with ipilimumab may offer more benefit than anti-PD-1 monotherapy for certain patients, e.g., those with BRAF-mutant melanoma, liver or brain metastases, rare melanoma subtypes (e.g., mucosal, acral, uveal), high lactate dehydrogenase (LDH) or high tumour burden. In an effort to reduce the significantly greater toxicity of the combination versus anti-PD-1 alone, nivolumab 3 mg/kg plus low-dose ipilimumab 1 mg/kg was investigated in the CheckMate 511 trial and shown to have similar survival outcomes to nivolumab 1 mg/kg plus ipilimumab 3 mg/kg with a significantly lower incidence of treatment-related toxicity [2]. This might be a more appropriate option for patients unable to tolerate higher doses of ipilimumab.

LAG-3 is a marker of T cell exhaustion, with exhausted CD8+ and CD4+ T cells progressively co-expressing multiple inhibitory checkpoints during chronic antigen stimulation [3]. LAG-3 is expressed immediately after PD-1 and blockade of both these inhibitory receptor pathways results in substantially greater reversal of T cell exhaustion compared to blockade of either alone. In the proof-of-concept RELATIVITY 020 study, the anti-LAG-3 agent relatlimab in combination with nivolumab showed activity in patients with advanced melanoma who were refractory to or relapsed on previous anti-PD-1/PD-ligand (L)1 therapy [4]. Responses were more likely in patients with LAG-3 expression ≥ 1% but PD-L1 expression did not appear to enrich for response. As first-line treatment in the phase II/III RELATIVITY 047 trial, median progression-free survival (PFS) was 10.1 months with nivolumab plus relatlimab versus 4.6 months with nivolumab alone in patients with previously untreated metastatic or unresectable melanoma [5]. Response rate was also improved in the combination arm. There were increased grade 3–4 adverse events with the combination compared to nivolumab monotherapy, but the toxicity burden of the combination was lower than that previously observed with nivolumab plus ipilimumab, with 21% of patients experiencing a grade 3–4 adverse event.

Strong T cell receptor (TCR) signalling results in higher LAG-3 expression, meaning that the effect of LAG-3 blockade is amplified in conditions of strong immunogenic signalling [6]. Conversely, LAG-3 expression is weaker under low immunogenic conditions with LAG-3 blockade consequently having a more limited effect. This may be one possible explanation for the greater activity of relatlimab as first-line therapy (43% response) compared to second-line treatment in patients refractory to anti-PD-1 therapy (13% response) [4, 5]. Consistent with this idea that earlier stages of disease have less T cell exhaustion and more TCR signalling, neoadjuvant treatment with nivolumab and relatlimab in combination resulted in a pathologic complete response (pCR) in 57% in patients with resectable clinical stage III/IV melanoma, with almost no grade 3–4 toxicity [7].

Given this, relatlimab in combination with nivolumab may be a better treatment option in patients who would otherwise be candidates for single-agent nivolumab or nivolumab plus low-dose ipilimumab. In addition, patients with or without BRAF-mutated melanoma, as well as those with acral and mucosal melanoma, a high tumour burden, or M1a/b baseline metastases, all benefit from nivolumab plus relatlimab versus nivolumab alone [5]. The effect of nivolumab plus relatlimab in patients with untreated melanoma brain metastases is currently being investigated in an ongoing trial.

Nivolumab plus relatlimab is superior to single-agent nivolumab across patient populations, with the added toxicity consistent with single-agent therapy and largely manageable. This combination should replace nivolumab plus low dose ipilimumab. Nivolumab plus higher-dose ipilimumab still has a role in special populations, e.g., patients with brain metastases, but even then only until data indicating otherwise become available. The use of nivolumab plus ipilimumab should be limited to second-line or salvage therapy, as in the SWOG 1616 trial which reported improved PFS and a higher ORR with nivolumab plus ipilimumab versus ipilimumab alone in patients with advanced melanoma refractory to anti-PD-1/PD-L1 treatment [8]. However, nivolumab plus relatlimab should be the preferred first-line option, sparing many patients the high-risk of ipilimumab-related toxicity. In the future, ipilimumab may still have a first-line role, but as part of a triplet combination with anti-PD-1 plus relatlimab, as will be investigated in a proposed SWOG trial.

### Omid Hamid: in favour of ipilimumab

Multiple trials across multiple years in various melanoma patient populations have proven the benefit of nivolumab plus ipilimumab over nivolumab alone. This includes in patients with liver metastases [1], uveal melanoma [9], mucosal melanoma [10] and BRAF-mutated melanoma [11] and represents the majority of patients with melanoma. After 7.5 years of follow-up in CheckMate 067, 48% of patients in the intent-to-treat population treated with nivolumab plus ipilimumab were still alive and one-third were progression-free [12]. Moreover, 77% of patients were alive and treatment-free (i.e., off-study treatment with no subsequent systemic therapy). The same claims cannot be made for in support of the combination of nivolumab plus relatlimab. Moreover, the combination of nivolumab plus relatlimab does not appear to provide the same benefit in all patients, with longer median PFS in patients with LAG-3 expression of ≥ 1% compared to patients with LAG-3 expression < 1% [5].

In a comparison of ipilimumab 3 mg/kg compared with ipilimumab 10 mg/kg in patients with advanced melanoma, the higher dose was associated with significantly improved OS but with more treatment-related adverse events [13]. However, previous experience gained in managing the immune-related side effects of ipilimumab meant that the toxicity of the higher dose was manageable. As such, the proven benefit of 48% survival at 7.5 years with nivolumab plus ipilimumab should outweigh any concerns about adverse events, most of which can effectively be managed.

**Fig. 1:**
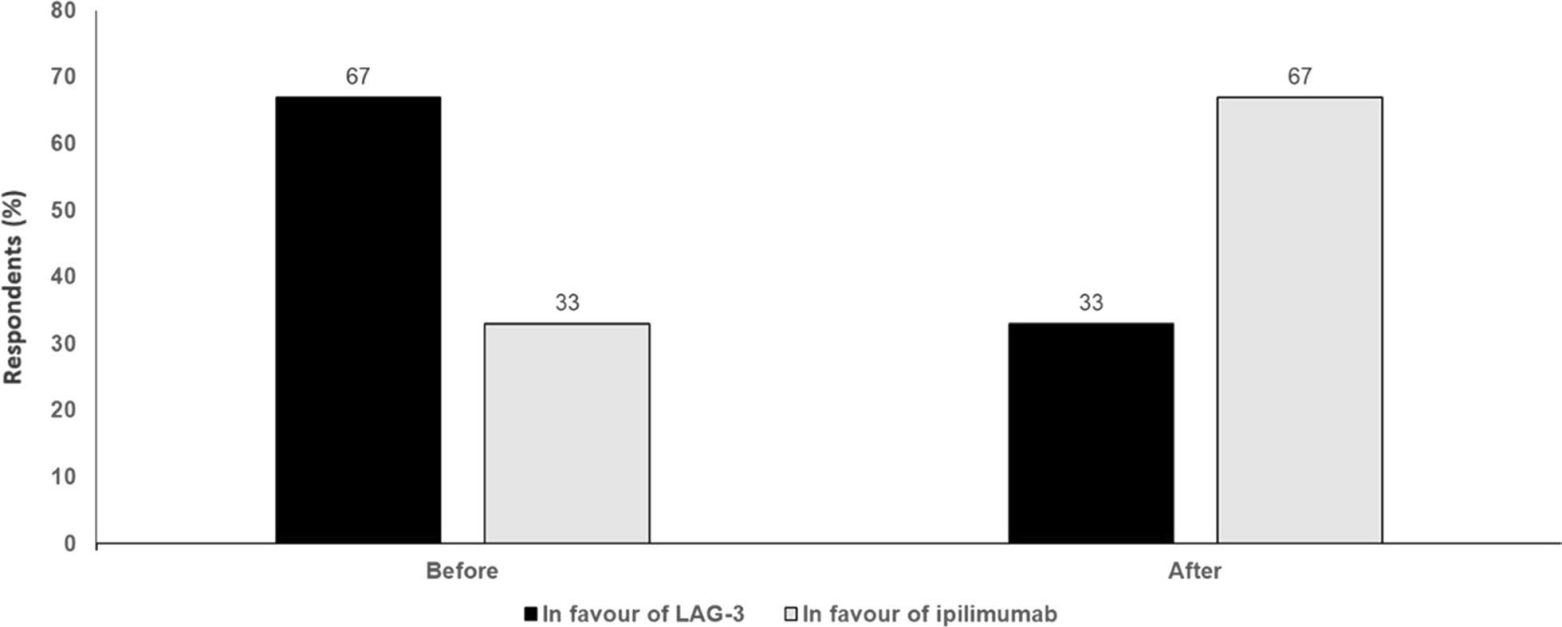
LAG-3 or Ipilimumab in combination with anti-PD-1. Audience response before and after debate

In patients with metastatic melanoma, choice of first-line therapy is critical given that the response to second-line therapy is never as good. For patients who do not respond to nivolumab and relatlimab, options for second-line treatment need to be considered. Most patients today have received adjuvant PD-1 treatment and so need a different option to nivolumab plus relatlimab. In the future, the best first-line option may be triplet or quadruplet therapy. For example, the interleukin-6 receptor blocking antibody, sarilumab, is being investigated in combination with ipilimumab, nivolumab and relatlimab in a phase II study of patients with unresectable stage III/IV melanoma. The discussion now is how to optimise checkpoint blockade. Second-line therapy will not be another checkpoint inhibitor but will involve an alternative approach, such as tumour-infiltrating lymphocyte (TIL)-based therapy or bispecific antibodies. These may be developed to have affinity to both cytotoxic T-lymphocyte-associated antigen (CTLA)-4 and PD-1 receptors, allowing higher dosing with less toxicity. Other bispecific antibodies include XmAb104, which simultaneously targets PD-1 and the immune co-stimulatory receptor, ICOS, and bavunalimab (formerly XmAb 22841), that simultaneously targets CTLA-4 and LAG-3. These two agents will be assessed in combination in a phase Ib/II study in patients with metastatic melanoma refractory to prior immune checkpoint inhibitor therapy and with or without central nervous system (CNS) involvement. Anti-LAG-3 has good tolerability, so will have a role as second-line therapy in combination with newer agents. Examples include ImmTAC® molecules, which are engineered to recognise intracellular cancer antigens with ultra-high affinity and selectively target these cancer cells via an anti-CD3 immune-activating effector function, and could be combined with LAG-3 inhibitors. Off-the-shelf therapeutics, e.g., allogeneic natural killer (NK) cells with a bispecific innate cell engager, may also be a well-tolerated option which could be combined with LAG-3 or PD-1 blockade (Fig. 1).

**Key points:**

- Clear long-term survival data with ipilimumab plus nivolumab indicates its superiority as first-line treatment.
- Anti-LAG-3 in combination with anti-PD-1 therapy may be a better treatment option in patients who would otherwise be candidates for single-agent nivolumab or nivolumab plus low-dose ipilimumab.
- Anti-PD-1 therapy plus ipilimumab may offer more benefit for certain patients, e.g., those with BRAF-mutant melanoma, liver or brain metastases, rare melanoma subtypes (e.g., mucosal, acral, uveal), high LDH or high tumour burden.
- More data is needed in relation to the long-term benefit of LAG-3 based therapy.

## Is anti-PD-1 monotherapy still acceptable as a comparator arm in clinical trials? Yes or No

### Claus Garbe: YES

In many centers, combined anti-PD-1 based immunotherapy is the most frequently used treatment regimen for patients with unresectable metastatic melanoma. This is most typically nivolumab plus ipilimumab in both Europe and the US, with nivolumab plus relatlimab also available in the US. However, anti-PD-1 monotherapy also remains a well-established regimen of choice.

Future clinical trials in metastatic melanoma are likely to have several major aims. These include increased efficacy of first-line treatments, identifying new immune checkpoint inhibitors with increased efficacy or reduced toxicity, and defining the therapeutic role of specific vaccine-based and cellular products. Choice of therapy in the control arm of any clinical trial must be an established standard of care treatment in metastatic melanoma, with acceptable toxicity and proven survival benefit (OS, or melanoma-specific survival [MSS]) as compared with previous treatments.

The two pivotal trials used to support the use of anti-PD-1 based combination therapy over monotherapy are the CheckMate 067 trial of nivolumab plus ipilimumab and RELATIVITY 047 trial of nivolumab plus relatlimab. In the CheckMate 067 trial, PFS rates at 6.5 years were 34% with nivolumab plus ipilimumab in combination versus 29% with nivolumab alone and 7% with ipilimumab alone [1]. Median OS in the combination group was 72.1 months, versus 36.9 months with nivolumab and 19.9 months with ipilimumab; however, the hazard ratio (HR) confidence intervals for the combination versus nivolumab alone crossed 1 (HR 0.84, CI 0.767–1.04) so was not significant. Results for MSS were similar, so no significant survival benefit of the combination over PD-1 monotherapy has been shown. In addition, grade 3 or higher toxicity of the combination was more than the sum of the toxicity in the two monotherapy arms.

In the RELATIVITY 047 study, median PFS was significantly improved with nivolumab plus relatlimab compared to nivolumab monotherapy (10.1 versus 4.6 months, HR for progression or death, 0.75 p = 0.006) [5]. Although this showed a significant benefit, it was not as favourable as the HR for the nivolumab plus ipilimumab combination versus nivolumab alone in CheckMate 067. One-year PFS rates were 48% with the combination and 36% with nivolumab. However, there are no data reported to date for OS or MSS. Grade 3–4 treatment-related adverse events occurred in 18.9% of patients in the combination group and 9.7% of patients in the nivolumab group.

In a post-hoc descriptive analysis of CheckMate 067, 6.5-year MSS rates were 56% with the combination, 48% with nivolumab and 27% with ipilimumab [1]. The combination was also associated with increased toxicity, with treatment-related grade 3–4 adverse events in 59% of patients treated with the combination compared with 21% treated with nivolumab and 28% treated with ipilimumab in the original 3-year analysis [14].

In conclusion, anti-PD-1 monotherapy remains an established standard of care in metastatic melanoma with a proven survival benefit over previous treatments, whereas any OS and MSS benefit of combination immune-checkpoint blockade over monotherapy is still unproven. Moreover, combination therapy is associated with a significant additional toxicity burden compared with monotherapy. As a result, single-agent anti-PD-1 treatment should still be considered an acceptable comparator arm in clinical trials.

### Jeffrey A. Sosman: NO

The time has now arrived that PD-1 blockade with nivolumab or pembrolizumab alone can no longer be considered a standard of care or an appropriate control arm for clinical studies in metastatic melanoma. The combination of nivolumab plus relatlimab resulted in significantly better PFS than nivolumab alone in the RELATIVITY 047 study and this was maintained from 1-year to 2-years of follow-up [5]. In addition, although median OS was not significantly different between the groups, OS curves were diverging in favour of the combination. Patients with PD-L1 expression of < 1% had greater benefit from the combination, with a HR for progression or death of 0.66, than those with PD-L1 expression ≥ 1, among whom median PFS was similar in the combination and monotherapy groups. Of note, the percentage of patients with progressive disease was also lower with the nivolumab plus relatlimab combination compared to nivolumab alone (30% versus 42%) and progression was seen at the first assessment (6 to 12 weeks) in more patients receiving monotherapy. Toxicity was increased with the nivolumab plus relatlimab combination, with treatment-related grade 3–4 adverse events in 21% of patients versus 11% with nivolumab alone. However, the toxicity of the combination arm was similar to that seen in earlier trials of single agent nivolumab and it may be that adverse events were underestimated in the monotherapy arm in this study.

In the neoadjuvant setting, nivolumab plus relatlimab resulted in a pCR rate of 57% and overall pathologic response rate of 70% among 30 patients with resectable stage III/IV melanoma [7]. The 2-year recurrence-free survival (RFS) rate was 92% for patients with any pathologic response, compared to 55% for patients who did not have a pathologic response. The 1- and 2-year OS rates for all patients were 93% and 88%. Based on these findings, the combination of nivolumab plus relatlimab appears to be more effective than neoadjuvant PD-1 monotherapy. Thus, even if anti-PD-1 combination and monotherapy have not been directly compared in a neoadjuvant clinical trial, the data so far suggest combination therapy would be a more appropriate control arm.

In a cross-trial comparison of nivolumab plus either relatlimab (RELATIVITY 047) or ipilimumab (CheckMate 067), the benefit:risk ratio, presented as the ratio of PFS improvement to toxicity × 100, favoured nivolumab plus relatlimab [15]. In another comparison, the PFS of both combinations were similar, but nivolumab plus relatlimab tended to show an earlier survival benefit and fewer treatment-related adverse events [16].

**Fig. 2:**
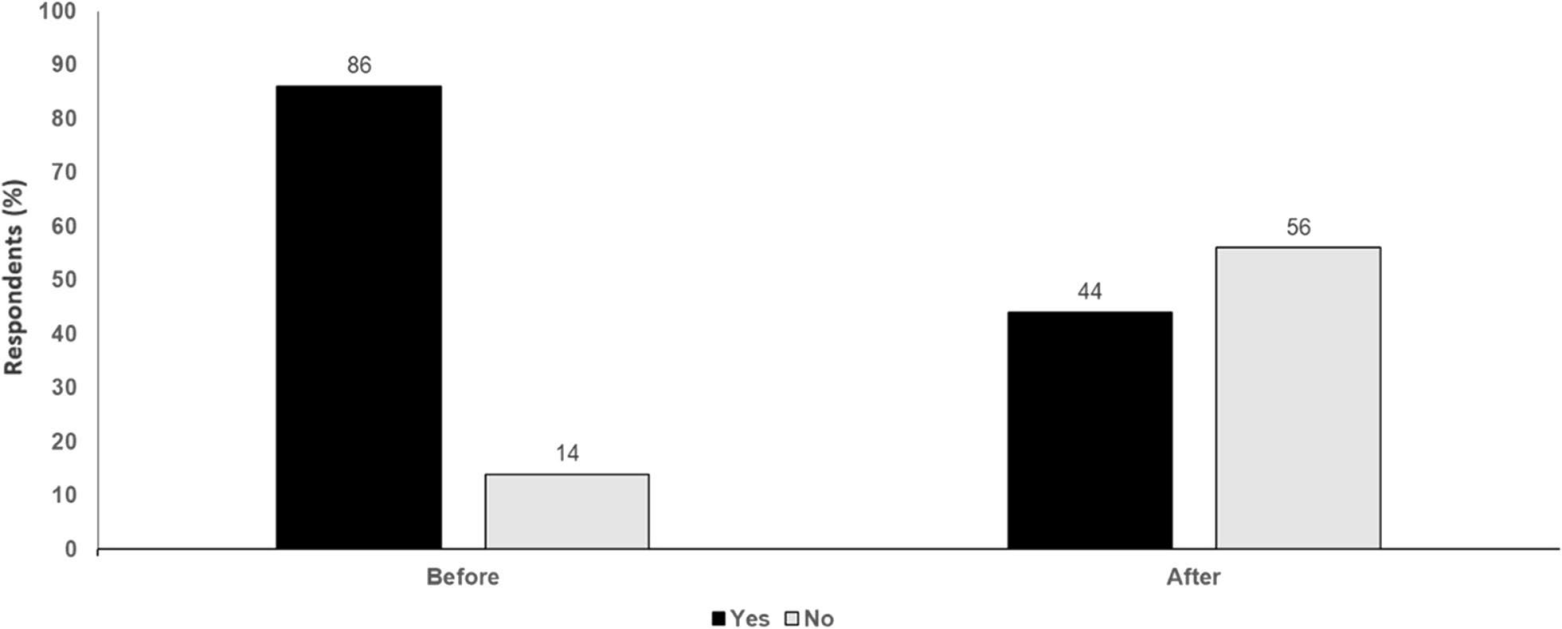
Is anti-PD-1 monotherapy still acceptable as a comparator arm in clinical trials? Yes or No. Audience response before and after debate

For future trials in melanoma, there should be no single agent anti-PD-1 arm as the single control arm. In both the metastatic and neoadjuvant settings, nivolumab plus relatlimab combination appears most active and less toxic than nivolumab plus ipilimumab, although the two combination approaches should be directly compared to make a strong definitive statement. A triplet combination of nivolumab/ipilimumab/relatlimab may become a future experimental arm in clinical trials. However, ultimately a better understanding of biomarkers is needed to help guide treatment choices among all these regimens in combination or as single agents (Fig. 2).

**Key points:**

- Choice of therapy in the control arm of a clinical trial must be an established standard of care, with acceptable toxicity and proven survival benefit.
- Anti-PD-1 monotherapy has a proven survival benefit over previous treatments, without the additional toxicity burden of combination therapy.
- In both the metastatic and neoadjuvant settings, nivolumab plus relatlimab appears most active and less toxic than nivolumab monotherapy, which may no longer be the gold standard of care.

## Is adjuvant treatment of melanoma still needed?

### Axel Hauschild: YES

Five-year survival rates for stage III melanoma in different cohorts range from 80% in stage IIIA to 30% in stage IIID, which justifies the use of adjuvant treatment. Studies of adjuvant therapy in high-risk melanoma (dabrafenib and trametinib in COMBI-AD and pembrolizumab in KEYNOTE 054) have shown HRs of 0.51–0.61 for reduced risk of relapse and 0.56–0.62 for distant metastases in stage III disease [17, 18]. These findings have also now been shown in stage IIB/C melanoma, with lower risks of recurrence (HR 042–0.64) and distant metastases (HR 0.47–0.64) with pembrolizumab or nivolumab in the KEYNOTE 716 and CheckMate 76K trials, respectively [19]. Moreover, no impairment in health-related quality of life has been observed in any adjuvant trials in melanoma. Positive OS data have also been reported for adjuvant therapy. OS in the nivolumab arm in CheckMate 238 versus the adjusted placebo arm in EORTC 18071, which assumes a post-recurrence survival increase of 63% in the placebo arm, show a 35% reduced risk of mortality with treatment [20]. In addition, the COMBI-AD trial shows a direct comparison, with 3-year OS rate of 86% in the dabrafenib plus trametinib group and 77% in the placebo group (HR for death, 0.57), although this did not cross the prespecified interim analysis boundary [21]. In a 4-year model for COMBI-AD, estimated cure rate was 54% in the dabrafenib plus trametinib arm compared with 37% in the placebo arm, a difference of 17% [22]. As such, there are data to suggest adjuvant treatment has a survival benefit. Adjuvant therapy also has good tolerability with a low frequency of treatment discontinuations, although there are some serious and sometimes irreversible immune-related adverse events that are difficult to predict and that occur in around 3% of patients.

Prevention of recurrence is considered to be more important than concerns of adverse events or quality of life by patient who are candidates for adjuvant melanoma treatment. In a real-world survey of over 900 patients, approximately 25% with stage III disease declined adjuvant therapy, with the most cited reasons in descending order of frequency being age, presence of comorbidities, fear of relapse, fear of adverse events and loss of quality of life [23]. However, acceptance of adjuvant therapy was high, with 75% of patients willing to be treated.

Alternative options to the current approach include improved selection of high-risk patients who will benefit from adjuvant treatment, e.g., through the use of nomograms, risk prediction scores and gene expression profiling (GEP). Another option is neoadjuvant therapy with or without adjuvant treatment. In the OpACIN-neo study, two cycles of neoadjuvant nivolumab plus ipilimumab had a pathologic response rate (pRR) of 77% [24]. In the PRADO extension cohort of the OpACIN-neo study, adjuvant therapy with nivolumab or dabrafenib plus trametinib improved outcomes in patients with no response to neoadjuvant checkpoint inhibition, with 2-year RFS and distant metastases-free survival (DMFS) rates of 71% and 76%, respectively [25]. In the randomized phase II SWOG 1801 trial, neoadjuvant pembrolizumab led to better event-free survival (EFS) than adjuvant pembrolizumab in patients with stage III–IV melanoma (HR 0.58) after a median follow-up of 14.7 months, with 2-year EFS rates of 72% and 49%, respectively [26]. OS also favoured neoadjuvant therapy, with an HR of 0.63, although this was not statistically significant. The neoadjuvant arm in this trial should more correctly be described as *“perioperative”,* since patients received treatment with pembrolizumab both before and after surgery, so it is not a pure neoadjuvant approach. These may be practice-changing data, with meaningful reductions in recurrence seen with just three cycles of pre-surgery pembrolizumab and no additional toxicity compared with conventional adjuvant therapy. It is unclear whether this may become a replacement for or an alternative to ipilimumab plus nivolumab.

Only around 20% of all patients with stage III melanoma have palpable lymph nodes or skin metastases and are therefore candidates for neoadjuvant treatment. In the future, there may be even fewer candidates due to more effective adjuvant stage II treatment. To date, there are no neoadjuvant trials in stage IIB/C melanoma. Adjuvant treatment remains an important option, although a neoadjuvant approach represents a valuable alternative for selected patients.

### Christian Blank: NO (and YES!)

The goal of adjuvant therapy is to improve the OS of curatively treated patients. However, even after 5 years of follow-up we have not seen a statistically significant OS benefit, according to prespecified boundaries, with adjuvant immunotherapy or targeted therapy in melanoma. This does not mean we should stop adjuvant therapy completely, but better selection of patients that will truly benefit is needed.

Adjuvant targeted or immunotherapy benefits less than 20% of patients with stage III melanoma, i.e., 80% of patents are treated for no clinical benefit but at a significant financial cost and exposure to adverse events. In COMBI-AD, the absolute difference in RFS rate at 4 years was 16% (4-year RFS of 54% with dabrafenib plus trametinib and 38% with placebo) [22]. Similarly, in KEYNOTE 054, 5-year rate of RFS was 55% with pembrolizumab versus 38% with placebo (17% absolute difference) [27]. Similar results were seen in both trials for DMFS (absolute differences of 12% at 4 years in COMBI-AD and 17% at 5 years in KEYNOTE-054).

An OS difference is also unlikely to be observed after even longer-term follow-up. Among patients with distant recurrence, approximately 20% will have distant metastases that can be salvaged with local therapy (radiotherapy or surgery) [28]. Response rate on nivolumab plus ipilimumab after anti-PD-1 is 40% versus 58% in treatment-naïve patients with low tumour burden and normal LDH. Of the remaining patients who progress on nivolumab plus ipilimumab, half have a BRAF mutation and early BRAF inhibitor therapy can achieve a 70% OS at 3 years. Based on these subsequent therapies, there will be no OS difference between the adjuvant and placebo arms at 5 years in COMBI-AD, KEYNOTE 054 or other future adjuvant trials. Thus, we will only see OS benefit if we shift to a better use of adjuvant therapy requiring baseline biomarkers to identify patients who will benefit from adjuvant therapy and treat only them.

The same applies in stage II melanoma, where even less absolute benefit is of only around 10% of patients seeing a RFS or DMFS benefit with adjuvant immunotherapy [29]. Without systemic therapy, patients with stage III melanoma have a 5-year OS of only 30–60% but RFS remains poor even with adjuvant therapy. A major reason for that the expected benefit of adjuvant therapy has not been observed in these trials is that patients with a worse prognosis were excluded. Adjuvant therapy is started 12 weeks after surgery and, in this time, 15–25% of patients progress and were excluded in all trials. These patients with early progression are those with the most aggressive melanomas, and likely would benefit most from a fast start of additional systemic therapy. If these patients were included, EFS of adjuvant therapy should be around 50% (currently reported 70% minus the 15–25%). This is indeed illustrated in the SWOG 1801 trial, which included such patients upfront, in which 2-year EFS in the adjuvant pembrolizumab arm was 49%, compared to 72% in the neoadjuvant arm [26]. As well as a significant EFS benefit with neoadjuvant over adjuvant therapy has been seen in this trial, the HR for OS (0.63) also favoured neoadjuvant, although is not yet significant.

Furthermore, the neoadjuvant arm involved 15 cycles of pembrolizumab after surgery, i.e., neoadjuvant plus adjuvant, and it is not known whether this is needed for all patients. In the PRADO trial, patients with major pathologic response (MPR) had 2-year RFS and DMFS rates of 93% and 98%, without therapeutic lymph node dissection (TLND) and adjuvant therapy [25].

Vice versa, adjuvant systemic therapy improved the RFS and DMFS rates of patients with no response to neoadjuvant checkpoint inhibition. In PRADO, 2-year RFS rate was 71% in non-responders, which compares with a 2-year RFS of 37% in non-responders, who did not receive adjuvant therapy in the OpACIN-neo study [30]. This absolute difference of 34% suggests that non-response to neoadjuvant immunotherapy may be a good biomarker identifying patients that will have an OS benefit from adjuvant therapy. This approach is being investigated in the response-driven phase III NADINA trial, in which patients with an MPR to neoadjuvant ipilimumab plus nivolumab will not receive adjuvant therapy, whereas patients without a response will receive adjuvant nivolumab or dabrafenib plus trametinib.

**Fig. 3:**
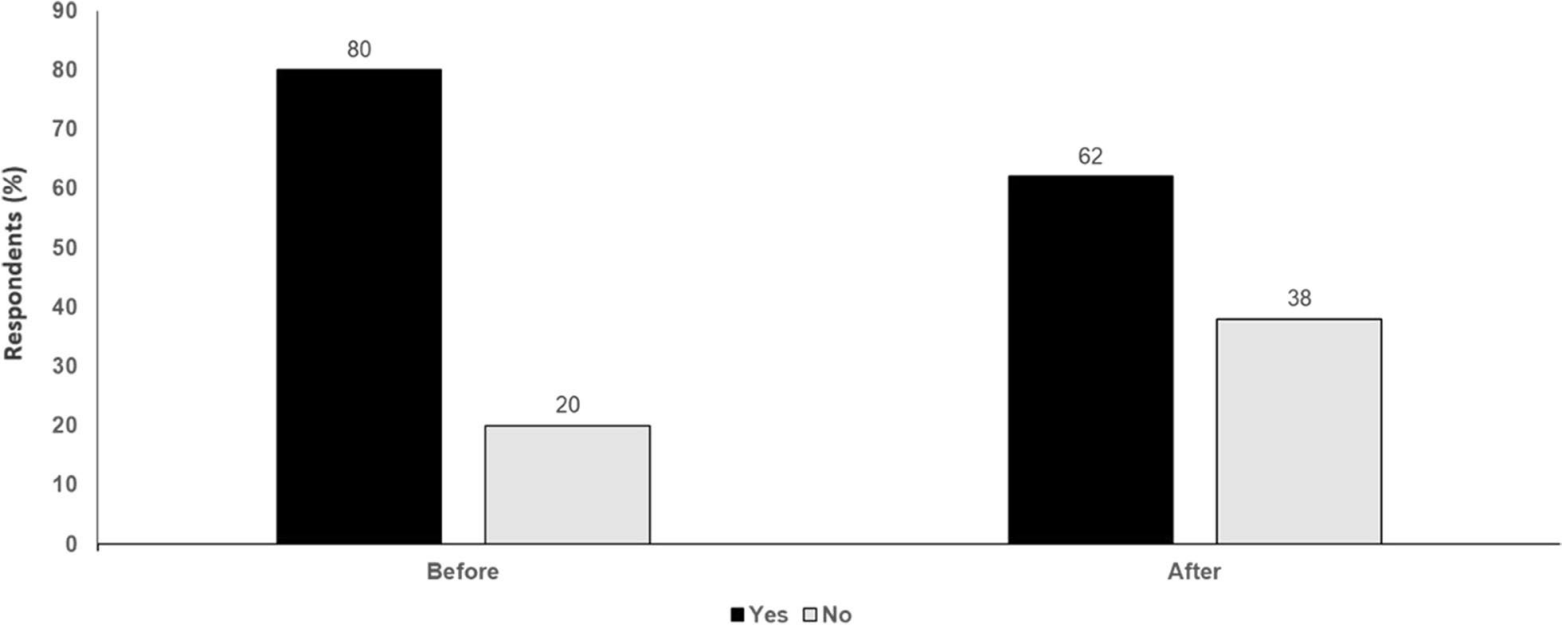
Is adjuvant treatment of melanoma still needed? Yes or No. Audience response before and after debate

In conclusion, adjuvant therapy for all patients with resectable stage II/III melanoma is not a valid option, because for these whole unselected cohorts there will be no OS benefit, and this at high financial and adverse event costs of patients treated for no benefit. A neoadjuvant approach is the way forward to solve this dilemma. Identifying non-responders after neoadjuvant therapy might become a strong biomarker for identifying patients with stage III disease who need adjuvant therapy. In stage II melanoma, a neoadjuvant approach still needs to be developed (Fig. 3).

**Key points:**

- Data suggest adjuvant treatment has a survival benefit with good tolerability in high-risk melanoma.
- Even after 5 years of follow-up we have not seen a statistically significant OS benefit with adjuvant immunotherapy or targeted therapy in melanoma so better selection of patients that will truly benefit is needed.
- Adjuvant treatment remains an important option, although a neoadjuvant approach represents a valuable alternative for selected patients.

## Stage II adjuvant therapy: is there space for locoregional approaches? Yes or No

### Alexander M. Eggermont and Jason J. Luke: YES

Stage II melanoma represents around 15% of patients with melanoma, with around one‐half of these patients having stage IIB or IIC disease and so being at higher risk of recurrence [31]. The number of patients with stage IIB/C melanoma is broadly similar to the number of patients who present with stage III melanoma. MSS for patients with stage IIB or IIC disease is similar to patients with stage IIIA/B disease. More initial recurrences in stage IIB/C melanoma are nodal or systemic rather than local/in-transit [32]. Relapses happen early in patients with stage II disease, as shown in the EA1697 phase III randomized trial of high dose interferon-α versus placebo [33].

Anti-PD-1 therapy reduces recurrence and distant metastasis. In the KEYNOTE 716 trial, adjuvant pembrolizumab significantly improved RFS and DMFS (both HR 0.64) versus placebo after 27 months of follow-up in patients with stage IIB or IIC melanoma [29]. Similarly, adjuvant nivolumab resulted in a statistically significant improvement in RFS in patients with completely resected stage IIB or IIC melanoma compared with placebo, with a 58% reduction in risk of death or recurrence compared with placebo [19]. However, the possible impact of adjuvant therapy on OS is unclear. Improved RFS corresponds with a similar improvement in DMFS; however, after diagnosis of distant metastases, salvage therapy will result in the curves coming together, with no difference in OS. Thus progression/recurrence-free survival 2 (PRFS2), which is defined as the time from initial random assignment to the second objective disease progression or recurrence, many be a better surrogate for OS. In the KEYNOTE 054 trial in stage III melanoma, treatment resulted in an improvement in PRFS2 with 5-year rates of 68% with adjuvant pembrolizumab versus 56% with placebo [27].

However, even without a proven OS benefit, adjuvant therapy may still be important. Although OS was rated the most important factor by patients when surveyed about attitudes to treatment decision-making, reduction of relapse was prioritized over risk of toxicity [34]. As such, reduced risk of recurrence should be considered as a significant quality of life benefit for patients and an important consideration when deciding treatment approaches.

In conclusion, resectable stage IIB/C melanoma has a significant risk of recurrence. Anti-PD-1 therapy improves RFS and DMFS and most likely also PRFS2. No OS benefit has been reported in any peri-surgical (neoadjuvant or adjuvant) anti-PD-1 trial but reduction in relapse is in itself considered a significant benefit by many patients.

### Janice M. Mehnert: NO

The proportion of patients with stage II melanoma who benefit from adjuvant therapy is limited and the vast majority with stage IIA/B disease do well without treatment; overtreatment is an issue given that only 12% with stage IIA disease, 18% with stage IIB and 25% with stage IIC benefit from adjuvant treatment [35].

Adjuvant therapy in stage II disease is a controversial topic and this can in part be attributed to the unpredictable nature of relapse, which may or may not be salvageable. Patients with stage IIB and IIC melanoma have a high risk of recurrence after 24 months with poor outcomes [36]. Pembrolizumab improved RFS versus placebo in patients with stage IIB/C melanoma in the KEYNOTE 716 study [29]. At a median follow-up of 27.4 months, median RFS was 37.2 months and the risk of recurrence remained lower with pembrolizumab versus placebo (HR 0.64). Somewhat unexpectedly, patients with T4b disease appeared to achieve less benefit from treatment than patients with T3b or T4a. Pembrolizumab also significantly improved DMFS versus placebo (HR 0.64, p = 0.0029). However, the pattern of distant metastases is important to consider, being largely driven by lung metastases which can be effectively treated at relapse. Other metastases which may be of greater concern, such as visceral or CNS, are still infrequent with or without treatment. This needs to be taken into consideration when deciding whether to offer adjuvant therapy to patients with stage II disease.

In KEYNOTE 716, 16% of pembrolizumab-treated patients had grade 3–4 treatment-related adverse events, which is not a trivial proportion. Similarly, grade 3–4 treatment-related toxicity occurred in 10% of nivolumab-treated patients in the CheckMate 76 K study [19]. Although some side effects may be manageable from a clinician’s perspective, they can still represent life-changing events for patients, e.g., hypothyroidism requiring endocrine therapy. In addition, other more obviously serious rare events, e.g., type 1 diabetes, can occur. These risks need to be considered given many patients will receive no benefit from therapy. Patient-centered considerations need to be our priority in treatment decision-making, with these including side effect profiles, treatment schedule and convenience of administration, life circumstances (e.g., fertility), concomitant medical conditions, and safety considerations, as well as cost. Adjuvant treatment is also not guaranteed to prevent relapse.

To date, we do not have data showing an OS benefit of adjuvant therapy in stage II disease. We do not know whether treating later will be equal in benefit to treating early. Those patients most likely to experience side effects cannot be predicted. Similarly, we do not have biomarkers for disease recurrence. More data on patterns of recurrence are required.

Alternatives to adjuvant therapy include careful observation or enrolment in clinical trials, although opportunities for study enrolment are limited. Targeted therapy may still be an option, as previously shown by a benefit with vemurafenib versus placebo in patients with stage IIC/IIIB melanoma [37]. Prospective data and biomarkers are needed to further assess this option, which is being investigated in the phase III COLUMBUS-AD trial of adjuvant encorafenib and binimetinib in high-risk stage II melanoma with a BRAF mutation.

**Fig. 4:**
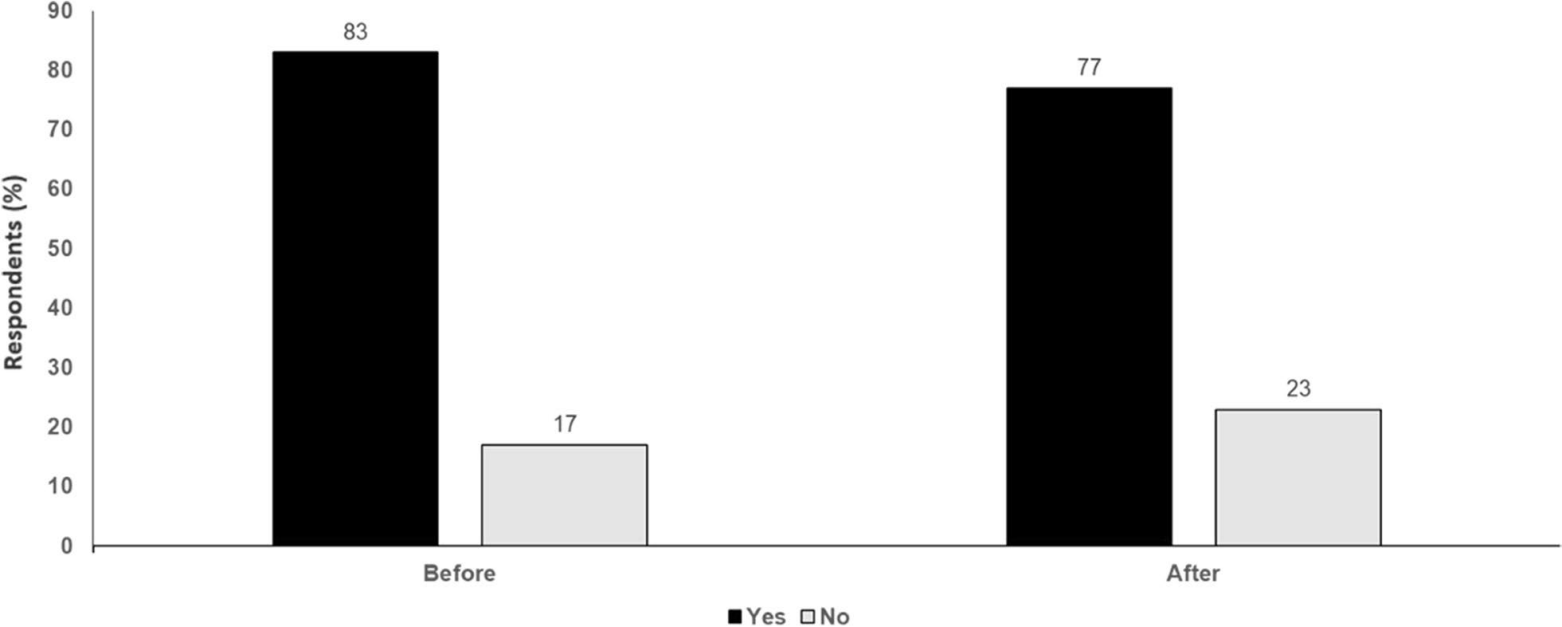
Stage II adjuvant therapy: is there space for locoregional approaches? Yes or No. Audience response before and after debate

Immunotherapy can offer the ‘gold star’ of a potential cure, but more data and rigorous biomarkers are needed to avoid overtreatment. It is essential that treatment goals and outcomes, including potential side effects and risks of recurrence with and without adjuvant therapy, are discussed with each individual patient (Fig. 4).

**Key points:**

- Anti-PD-1 therapy improves RFS and DMFS in resectable stage IIB/C melanoma.
- Although no OS benefit has been reported in any peri-surgical (neoadjuvant or adjuvant) anti-PD-1 trial, reduction in relapse is in itself a significant benefit for many patients.
- However, the vast majority patients with stage IIA/B disease do well without treatment and more data and rigorous biomarkers are needed to avoid overtreatment.

## Any role for surgery?

### Jeffrey E. Gershenwald: YES

The majority of patients diagnosed with early-¯stage melanoma are at low likelihood of relapse and will never require treatment beyond the primary site. A wide excision is recommended with margins based on the primary tumour thickness [38–40]. Ongoing efforts to de-escalate margins of excision, as is being investigated in the SWOG 2015 MelMart-2 study (NCT03860883) in which patients are randomised to wide excision with a 1 cm or 2 cm margin, does not constitute “no” surgery.

The second Multicenter Selective Lymphadenectomy Trial (MSLT-II) reported that CLND did not result in a MSS benefit versus observation in patients with melanoma and sentinel-node metastases [41]. This resulted in a paradigm shift away from CLND. Nodal only failure was lower with CLND (1.3% versus 7.7% with observation) but lymphedema was observed in 24% of the patients receiving CLND compared with 6.3% in the observation group. Sentinel lymph node biopsy (SLNB) is recommended in patients with tumour thickness ≥ 1 mm (or ≥ 0.8 mm with additional histological risk factors) [38–40]. Despite international guideline-driven recommendations, it has been suggested by some that since adjuvant therapy is approved for stage IIB/C and stage III patients, SLN biopsy will not inform clinical decision-making. In MSLT-I, SLN biopsy-based staging of intermediate-thickness or thick primary melanomas provided prognostic information of which patients with nodal metastases may benefit from immediate CLND [42]. In patients with thick melanomas (defined as > 3.50 mm tumor thickness in MSLT-I) who underwent wide excision and SLNB, around one-third had a positive SLN biopsy at time of wide excision, and by 10 years, the overall incidence of regional metastasis (SLNB positive + clinically detected recurrence after negative SLNB) was 42%. In the wide excision only arm, a nearly identical 41.4% of patients developed clinically detected recurrence after regional node basin observation at 10 years. Remarkably, approximately 25% of patients in the wide excision only arm developed clinical evidence of regional nodal metastasis within 1 year, i.e., during the time in which adjuvant therapy might be given, and suggests that a significant fraction of patients may develop clinical regional nodal recurrence during adjuvant therapy if SLNB is not performed.

In long-term follow-up of MSLT-II, 80.2% of basins were free of nodal recurrence at 10 years in the observation arm, supporting that there is a therapeutic value of SLNB in patients with melanoma [43]. Risk factors for in-basin recurrence were age ≥ 50 years, ulceration, Breslow thickness > 3.5 mm, non-axillary basin, and tumour burden of maximum diameter ≥ 1 mm and/or metastasis area of ≥ 5%.

Risk prediction tools which use conventional biomarkers, such as the Melanoma Institute Australia Prediction Tool for Sentinel Node Metastasis Risk (https://melanomarisk.org.au/SNLForm) [44], or combined use of clinicopathological factors and GEP may be useful. Further refinement of staging based on sentinel node data may help also stratify risk. For example, among 3607 patients with clinical T1b and T2a primary cutaneous melanoma, SLN maximum tumour dimension ≥ 0.3 mm significantly increased risk versus < 0.3 mm, with 5-year disease-specific survival rates of 80% and 94% respectively (HR 1.26; p < 0.0001) [45]. Low-risk (< 0.3 mm) stage IIIA patients had similar survival to patients with IB disease. The authors concluded that adjuvant therapy (or clinical trial) may be considered for high-risk IIIA patients, whereas similar treatment to stage IB should be considered for patients with low risk IIIA disease.

In a retrospective study of 1377 patients with pT1–pT4b primary cutaneous melanoma, the optimal maximum tumour deposit size cut-point was 0.7 mm for the pT1b-pT4a SLN-positive subgroups, but there was no cut-point for SLN-positive patients with pT4b melanoma [46]. Nodal risk categories were developed using the 0.7 mm maximum tumour deposit size cut-point and extracapsular spread status. Patients with maximum tumour deposit size ≤ 0.7 mm and no extracapsular spread were low risk, patients with maximum tumour deposit size > 0.7 mm and no extracapsular spread were intermediate risk, and patients with high extracapsular spread were high risk irrespective of maximum tumour deposit size.

Adjuvant therapy reduces risk of recurrence and improves survival following initial treatment and in whom there is no evidence of disease. However, clinical benefit is only possible if residual sub-clinical disease exists. It is important to recognize that absolute benefit is a function of hazard ratio and overall risk of recurrence. Moreover, since toxicity may occur in any patient who receives adjuvant therapy, with the potential for lifelong sequalae, it is critically important to weigh the potential benefits, risks, and alternatives of any potential treatment strategy. SLNB is an important staging technique that improves regional control, may have a therapeutic benefit, and helps clinicians and patients make more informed treatment decisions.

In the metastatic setting, surgery may be selectively deployed, with long-term survival following metastasectomy in highly selected patients having a limited but important role, while the consolidation or resection of resistant clones in the setting of systemic therapy can help mitigate/palliate symptoms, such as pain, bleeding, or obstruction, and facilitate considerations for additional systemic and/or other approaches.

To conclude, despite exciting times in this era of effective systemic therapy, there is still a significant role for surgery across the continuum of melanoma that continues to evolve and is unlikely to completely disappear.

### Alexander M. Eggermont: NO

Clearly surgery is required for resection of primary melanoma. However, beyond this, the number of surgical interventions in melanoma could be reduced by up to 80% in Europe over the next 5 years. Importantly, lymphocytes are primed and ‘educated’ in the lymph nodes so their removal should be avoided where possible.

Wide excision for primary cutaneous melanoma is recommended with margins based on the primary tumour thickness [37]. However, there is no evidence to support the use of wide local excision for primary melanoma that has been completely excised on diagnostic excision biopsy [47].

Positive sentinel node staging used to provide an indication for CLND, although this is no longer the case since the MSLT-II and DECOG trials showed no OS benefit [41, 48]. A positive SLNB also provided an indication for adjuvant therapy in stage III melanoma, with a 40–50% reduction in relapse. However, this is no longer necessary since the KEYNOTE-716 and CheckMate 76K studies in stage IIB/IIC melanoma, the results of which will lead to a reduction of SNLB of about 50% [49, 50]. The next development will be the use of GEP combined with clinical features (e.g., Breslow thickness), especially for patients with stage IB-IIA disease, to help identify which patients will relapse. This type of profiler may make SNL biopsy redundant [50, 51]. Biomarker-based risk stratification is being investigated in the NivoMela trial of adjuvant treatment of stage IIA-C melanoma, in which patients with a negative SLN biopsy and high-risk GEP score are randomized to nivolumab or observation while low-risk GEP score patients are not randomized but are observed during follow-up. Using archival specimens from patients with stage I/IIA melanoma, clinicopathologic (CP)-GEP identified a high-risk patient group with significantly worse 5-year RFS than the low-risk patient group (74% versus 89%, HR, 2.98, p < 0.0001) [51]. CP-GEP also identified high-risk SLN biopsy-negative candidates for adjuvant therapy. RFS benefit translated into DMFS and MSS benefits. This test was validated in 424 patients with stage I/IIA melanoma, with 5-year RFS rates of 77.8% in CP-GEP high risk and 93% in CP-GEP low risk patients [52]. CP-GEP identified 6 out of 7 relapses in patients who did not receive SLN biopsy. This profiling will largely replace sentinel node staging as a test on the primary melanoma.

With regard to TLND, this has largely been replaced by neoadjuvant immunotherapy, as shown by the PRADO trial in which patients with MPR in their index lymph node after neoadjuvant nivolumab and ipilimumab did not receive TLND or adjuvant therapy; 2-year RFS was 93% in these patients [25]. Neoadjuvant checkpoint inhibition is thought to be superior to adjuvant due to induction of a larger and broader immune repertoire and induces high pathological response rates associated with prolonged RFS [53]. This approach may lead to more cures, shorter treatment cycles and less surgery.

Further surgical uses include resection of In-transit metastases, which can mostly be treated with immunotherapy (checkpoint inhibitors or local talimogene laherparepvec) although there is a still an occasional need for excision of oligometastatic disease. Distant metastases can be treated with neoadjuvant checkpoint inhibition, with only around half needing resection. Escape lesions are very rare and will be treated by organ specialist surgeons. Palliative interventions for local control may be sometimes needed but are also rare; neoadjuvant immunotherapy and radiation therapy may also have a role in these patients.

**Fig. 5:**
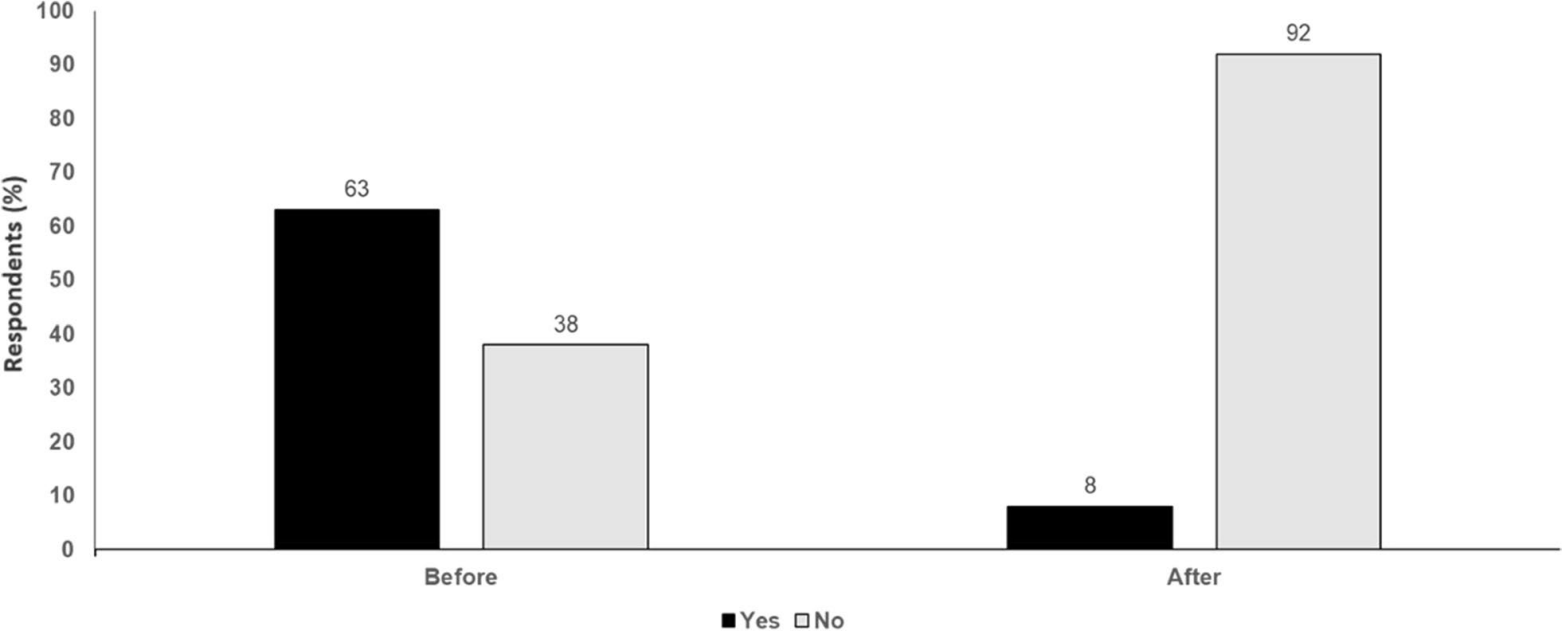
Any role for surgery? Yes or No. Audience response before and after debate

To conclude, melanoma surgery will be reduced by > 60% in the short term and by > 80% in the next 5 years, being replaced by (neo)adjuvant therapies and the use of SLNB replacement technologies. Neoadjuvant immunotherapy will replace the standard adjuvant therapy model and reduce TLND, metastasis resections, and SLNB [49, 50] (Fig. 5).

**Key points:**

- There is still a significant role for surgery across the continuum of melanoma that continues to evolve and is unlikely to completely disappear.
- Although required for resection of primary melanoma, beyond this the number of surgical interventions in melanoma could be significantly reduced over the next 5 years.
- Surgery will be replaced by (neo)adjuvant therapies and the use of SLN biopsy replacement technologies.
- Neoadjuvant immunotherapy will replace the standard adjuvant therapy model and reduce TLND, metastasis resections, and SLN biopsy.

## Conclusions

The Melanoma Great Debate included the presentation of counterpoint views from leading experts on five contemporary clinical issues in the management of melanoma. Given the format and nature of the debates, presentations were not intended as a rigorous and/or systematic assessment of the field but instead allowed the opportunity to highlight some important questions and current controversies. These debates are obviously more nuanced than the simple for or against/yes or no format encourages; however, it is hoped that these discussions can help focus attention on these issues, stimulating further research needed to improve our understanding of different therapeutic approaches.

## Data Availability

Not applicable.

## Abbreviations

CLND: Completion lymph-node dissection
CNS: Central nervous system
CP: Clinicopathologic
CTLA-4: Cytotoxic T-lymphocyte-associated antigen-4
DMFS: Distant metastases-free survival
EFS: Event-free survival
GEP: Gene expression profile
HR: Hazard ratio
LAG-3: Lymphocyte-activation gene-3
LDH: Lactate dehydrogenase
MLST: Multicenter Selective Lymphadenectomy Trial
MPR: Major pathological response
MSS: Melanoma-specific survival
NK: Natural killer
ORR: Objective response rate
OS: Overall survival
pCR: Pathological complete response
PD-1: Programmed death-1
PD-L1: Programmed death ligand-1
PRFS2: Progression/recurrence-free survival 2
PFS: Progression-free survival
RFS: Recurrence-free survival
SLNB: Sentinel lymph node biopsy
TCR: T cell receptor
TIL: Tumor-infiltrating lymphocytes
TLND: Therapeutic lymph node dissection
